# Rapid Determination of Several Biogenic Amines in Cold-Chain Fish Samples by Portable Ion Trap Mass Spectrometry with Nano-Electrospray Ionization

**DOI:** 10.3390/foods15101802

**Published:** 2026-05-19

**Authors:** Jianxin Wu, Xiaotong Ma, Zongyi Wang, Ying Wei, Yuting Liu, Jiaqian Men, Wenyu Ma

**Affiliations:** Beijing Key Laboratory of Agricultural Product Detection and Control for Spoilage Organisms and Pesticides, College of Food Science and Engineering, Beijing University of Agriculture, Beijing 102206, China; 15011216964@163.com (J.W.); m13095157604@163.com (X.M.); proudwy@126.com (Y.W.); 13020087674@163.com (Y.L.); 15383291685@163.com (J.M.); mawy2872@163.com (W.M.)

**Keywords:** biogenic amine, nano-electrospray ionization (nESI), portable ion trap mass spectrometry, cold-chain fish, eligibility fast screening

## Abstract

A novel method was developed for the rapid determination of five biogenic amines (BAs)—histamine (HIS), tyramine (TYR), cadaverine (CAD), spermidine (SPD), and spermine (SPM) in cold-chain fish by portable ion trap mass spectrometry with nano-electrospray(nESI) ionization. Samples were homogenized and extracted with aqueous solution containing 1% (*v*/*v*) formic acid and 80% (*v*/*v*) acetonitrile. With HIS-d4 as an internal standard, the sample solutions were directly injected with the nESI injection device and detected by a portable ion trap mass spectrometer at MS/MS detection mode. The results showed good linearity in the invested range of 0.2 (or 0.5)–10 μg mL^−1^ with R^2^ > 0.992, The limit of detection (LODs) and limits of quantification (LOQs) for HIS were less than 1.5 mg/kg and 4.0 mg/kg, respectively; the LOD and LOQ for other four BAs were less than 4.0 mg/kg and 12.5 mg/kg, respectively. Recoveries at three fortified levels ranged from 84.26% to 106.6% with relative standard deviations between 4.56% and 13.84%. With the safety limits of HIS as the concentrations of concern, this method demonstrated excellent performance when applied to the eligibility fast screening of HIS in cold-chain fish. The study provided a valuable methodological reference for the rapid detection of BAs in food.

## 1. Introduction

Biogenic amine (BA) is a class of basic nitrogenous compounds of low molecular mass in foods produced mainly by decarboxylation of free amino acids or aldoketo amination reactions [1,2]. Low levels of BAs in the body have important physiological functions such as growth regulation, neurotransmission, and inflammatory mediators [3,4], but consumption of foods with high levels of BA can lead to toxic reactions such as respiratory distress, elevated blood pressure, vomiting, and headache [5,6]. In addition, BA in foods can react with nitrites to convert them into carcinogenic nitrosamines [7,8]. Usually, fresh aquatic products contain low levels of BAs, but due to improper storage and transportation, these protein- and amino acid-rich aquatic products are prone to producing large amounts of BAs [9,10], which affects the quality of the products. As a result, BA has also become one of the key indicators for assessing food safety and freshness in many countries. There is no uniform international limit standard for BA in food, and the existing regulatory limits mainly focus on fish and their products, and the BAs involved are mainly histamine (HIS) and tyramine (TYR), which have the most toxic effects [11,12]. For example, the U.S. Food and Drug Administration (FDA) stipulates a limit level of 50 mg/kg for HIS and no more than 100 mg/kg for TYR, while in China, only a limit for HIS in aquatic products has been set, which stipulates a limit level of 400 mg/kg in high-histamine fishes, such as mackerel, tuna and skipjack tuna, and 200 mg/kg in other seawater fishes.

Currently, the analytical methods reported for BAs mainly include high-performance liquid chromatography (HPLC) using different kinds of detectors [13,14], gas chromatography-mass spectrometry (GC-MS) [15], high-performance liquid chromatography-tandem mass spectrometry (HPLC-MS/MS) or ultra performance liquid chromatography-tandem mass spectrometry (UPLC-MS/MS) [16,17], capillary electrophoresis (CE) [18] and ion mobility spectrometry[IMS] [19]. Among them, CE and IMS are less used in routine food analysis; HPLC and GC-MS both require pre-column or post-column derivatization, which is inconvenient; the advantages of HPLC-MS/MS or UPLC-MS/MS in solving the tasks of qualitative and quantitative analysis of micro- and trace multicomponent targets in complex matrices are very prominent, in terms of BAs, derivatization-free detection could be achieved, so it has been more widely used in the detection of BAs in food, but there are problems such as relying on large-scale equipment, high detection cost and inability to realize on-site detection.

Ambient ionization mass spectrometry (MS) is a rapidly developing ionization technique from the 2000s, which can complete the analysis of samples without or only with simple sample pretreatment and has the advantages of fast analysis speed, simplicity and high efficiency [20,21,22]. A large number of ambient ionization techniques have been reported and applied in the field of food and drugs to date [23,24]. In recent years, with the rapid development of mass spectrometry technology, the combination of ambient ionization technology and portable mass spectrometry has realized the miniaturization of the instrument, while still retaining the advantages of qualitative and reliable mass spectrometry, and also enabling rapid detection. The paper capillary spray (PCS) [25,26,27] and nanoelectrospray (nESI) technique [28] are two of the better-developed ambient ionization techniques used in portable mass spectrometers. The PCS technology combines a metal capillary with a paper substrate by loading solid or liquid samples onto the surface of the paper substrate, eluting them with a suitable solvent, and entering the capillary for electrospraying them, which improves spraying stability and sensitivity compared to paper spraying technology. The nESI technique loads a liquid sample into a glass capillary (lined with a metal wire) for electrospraying; Compared with PCS, nESI provides higher sensitivity, better reproducibility and larger sample loading capacity, which is more conducive to quantitative analysis. Both techniques are characterized by easy operation, high detection efficiency, and on-site real-time determination in conjunction with portable mass spectrometry, but few studies have been reported on analyzing BAs in cold-chain fish products. Therefore, it is of great value to apply this technique to the quality evaluation and monitoring of cold chain fish products.

In this study, it is proposed to nESI ionization combined with a small portable ion trap mass spectrometer to establish and validate a novel method for the rapid detection of five types of BAs, namely, HIS, TYR, cadaverine (CAD), spermine (SPM) and spermidine (SPD), in cold-chained fish products, which could provide a valuable technology reference for the rapid screening and on-site detection of BAs content in cold-chained fish products.

## 2. Materials and Methods

### 2.1. Chemicals and Reagents

The standards of HIS, CAD, TYR, SPM and SPD were purchased from Sigma Aldrich Shanghai Co. (Shanghai, China). Histamine-d4 (HIS-d4) was purchased from Tianjin Alta Technology Co. (Tianjin, China). HPLC-grade acetonitrile, methanol, formic acid and n-hexane were purchased from Beijing Myriad Technology Co. (Beijing, China).

### 2.2. Standard Solution Preparation

Stock solutions of each BA at 1.0 mg mL^−1^ and HIS-d4 at 0.1 mg mL^−1^ were prepared in methanol and were stored frozen between −12 °C and −18 °C. Intermediate standard solutions of the mixture of five BAs at 100 μg mL^−1^ and the HIS-d4 internal standard working solution at 10 μg mL^−1^ were prepared by diluting the stock solutions with methanol, respectively. The calibration standard solutions of the mixture of the five analytes (0.2, 0.5, 1, 2, 5 and 10 μg mL^−1^) were prepared by diluting the intermediate standard solutions with an aqueous solution containing 1% (*v*/*v*) formic acid and 80% (*v*/*v*) acetonitrile.

### 2.3. Sample Preparation

About 200 g of the edible portion of the was taken from each fish sample after de-boning and was fully crushed in a sample pulverizer (Midea MJ-WBL2531H, Guangdong Midea Living Electrical Appliances Manufacturing Co., Ltd., Foshan, China). The sample extraction was performed following the method described by WU et al. [29] with appropriate modifications and briefly summarized as follows: 2.0 g (accurate to 0.0001 g) of the sample was crushed and weighed into a 50 mL centrifuge tube, and 10 mL of aqueous solution containing 1% (*v*/*v*) formic acid and 80% (*v*/*v*) acetonitrile was added. Then, the sample in the tube was homogenized using a homogenizer (FSH-2 Adjustable High-speed Homogenizer, Changzhou Guohua Electric Co., Ltd., Changzhou, China) at a speed between 9500 and 10,500 r/min for 30 s. After that, the tube was put into the centrifuge (Eppendorf Centrifuge 5180 R, Eppendorf AG, 2231 Hamburg, Germany) and centrifuged at 8000 rpm for 5 min. The supernatant was transferred to a 25 mL volumetric flask. The blade head of the homogenizer was rinsed for 30 s at same rotational speed with another 50 mL centrifuge tube containing 10 mL of the aqueous solution containing 1% (*v*/*v*) formic acid and 80% (*v*/*v*) acetonitrile, and the rinsate was then poured into the original tube containing the sample residue for a second extraction by vortexing the mixture for 30 s and centrifuging again at 8000 rpm for 5 min; the supernatant from the second extraction was also combined into the same volumetric flask and was diluted to the volume with the aqueous solution containing 1% (*v*/*v*) formic acid and 80% (*v*/*v*) acetonitrile. Then, 2 mL of the sample extraction solution was transferred into a 10 mL centrifuge tube, and 3 mL of n-hexane was added. The tube was vortexed for 30 s, then left to stand until fully stratified, and the upper layer of n-hexane solution was discarded. The lower layer sample extraction solution was filtered through a 0.22 µm syringe filter and collected into a 2 mL vial, ready for analysis.

### 2.4. Portable Ion Trap Mass Spectrometry Analysis

#### 2.4.1. Instrumentation and Analysis Conditions

The rapid determination was carried out using a CELL portable ion trap mass spectrometry system on the MS/MS detection mode with PMS Client Pro analytical software and the simple, small nESI injection device containing an injection base and a nano-spray needle (Beijing Qingpu Analytical Instrument Co., Ltd., Beijing, China). The analytical parameters of each analyte are shown in Table 1.

#### 2.4.2. Operating Procedures

A volume of 100 μL of the final sample extraction solution and 20 μL of internal standard working solution were accurately pipetted into a 2 mL centrifugal tube and mixed by vortexing, then not less than 20 μL of the mixing sample solution was pipetted into the nano-spray needle of the nESI injection device. Next, the nESI injection device was inserted into the injection port of the small mass spectrometer and MS detection was performed. For the series of mixture calibration standard solutions, the detection was carried out with the same procedures.

### 2.5. Method Validation

#### 2.5.1. Linearity, Limit of Detection (LOD), Limit of Quantification (LOQ), Accuracy and Precision

Linearity was evaluated by injection of the calibration standard solutions and least-square regression between the concentration ratios of analytes to the internal standard HIS-d4 and the ratios of their response. The LOD and LOQ were estimated based on the quantitative results of the actual samples or the samples fortified with analyte at the low level, with a signal-to-noise ratio of ≥3 and ≥10 as criteria, respectively. The accuracy and precision were evaluated through analyzing the samples fortified with each analyte at three concentrations (near LOQ, medium, and high) and calculating the recoveries and relative standard deviations (RSDs).

#### 2.5.2. Threshold and Positive Detection Rate

Threshold is the value that determines whether the detection result exceeds the concentration of concern when a quantitative analytical method is used for the purpose of an eligibility screen. It was calculated according to the determining results of multiple replicate samples spiked with the analyte at the same level of concentration of concern. The calculation equation was as Formula (1).

(1) $$Threshold=\bar{x}-t_{\alpha }(f)\cdot \frac{S}{\sqrt{n}}$$

where $\bar{x}$ is the average result of the multiple independent determinations; $t_{\alpha }(f)$ is the value of the t-distribution when the significance level is *α* (here, *α* = 0.05) and the degree of freedom is *f* (here, *f* = *n* − 1); *S* and *n* are the standard deviation and number of independent repeated determinations, respectively.

For a concentration of concern, if the determining result of the analyte was above the threshold, the sample was recorded as a positive sample for the analyte, and if that was below the threshold, the sample was recorded as a negative sample for the analyte. Series gradient spiked samples with analyte concentrations close to (below, above, and equal to) the concentration of concern were prepared with 20 replicate samples for each gradient level. The analyte was determined in those samples, and positive detection rates were calculated at each fortified level. The positive detection rate, calculated according to Formula (2).

(2) $$positive\;detection\;rate\;=\;\frac{n}{N}\times 100\%$$

where *n* is the number of positive samples at a level; *N* is the total number of samples at the level.

The concentration level at which the positive detection rate is at least 95% is defined as the minimum reliable detection level for positive samples; the concentration level at which the positive detection rate is at most 5% is defined as the maximum reliable detection level for negative samples.

## 3. Results and Discussion

### 3.1. Mass Spectrometry Detection

**Figure 1:**
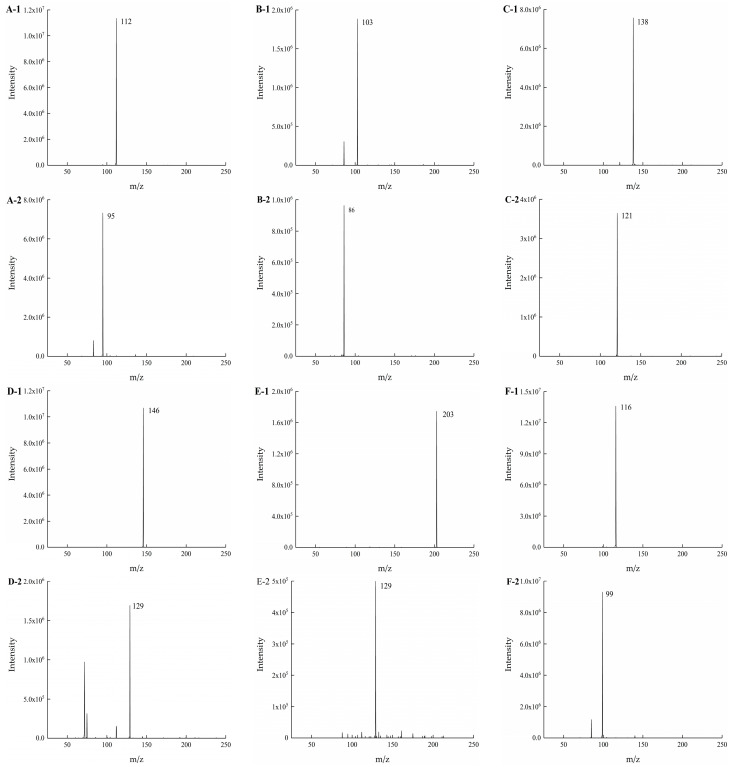
The primary mass spectra ((**A-1**) HIS, (**B-1**) CAD, (**C-1**) TYR, (**D-1**) SPD, (**E-1**) SPM and (**F-1**) HIS-d4) and secondary ((**A-2**) HIS, (**B-2**) CAD, (**C-2**) TYR, (**D-2**) SPD, (**E-2**) SPM and (**F-2**) HIS-d4) mass spectra.

Firstly, single standard solutions of each analyte at an injection concentration of 10 μg/mL were injected for analysis. The [M + H]^+^ ions of each target compound were selected as precursor ions for full-scan MS acquisition with a collision energy of 0 eV. Taking the maximum response intensity as the evaluation index, the ionization voltage and injection time were optimized to determine their optimal values. Subsequently, MS/MS scanning was performed at different collision energies to screen the optimal fragment ions and their corresponding collision energies. The optimized mass spectrometry parameters are summarized in Table 1. The mass spectra of the five target BAs and the internal standard HIS-d4 under the optimized conditions are shown in Figure 1. The sample solvent is another factor that affects the performance. Although multi-stage ion trap mass spectrometry can provide better selectivity, the detection sensitivity decreases accordingly. For the BAs compounds with small molecular masses ranging from 103 to 203 Daltons, here, the experiments showed that MS/MS detection not only ensured good selectivity but also maintained high sensitivity. Therefore, MS/MS detection was used.

**Figure 2:**
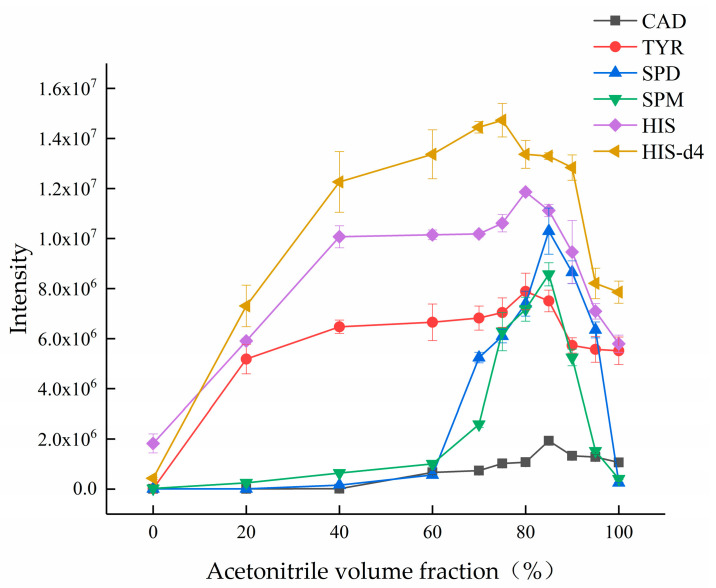
Effect of different acetonitrile volume fractions on the response values of analytes.

Sample solvent is another factor that affects the sensitivity of mass spectrometry detection. To better align the MS detection with the sample pretreatment process, in addition to adding 1% (*v*/*v*) formic acid to the solvent [29], the experiment further investigated the effect of different acetonitrile-water ratios. The results showed that when the acetonitrile ratio was between 70% (*v*/*v*) and 90% (*v*/*v*), the ion signals of the target compounds were relatively stronger; outside this range, the signal decreased significantly (see Figure 2). This is primarily because acetonitrile, as an aprotic solvent, is not conducive to the ionization of target compounds when its ratio is too high. Conversely, when the ratio is too low, the volatility of the solvent decreases, leading to poor nebulization efficiency. Therefore, the acetonitrile ratio in the final sample solution was controlled within this range. In this study, the aqueous solution containing 1% (*v*/*v*) formic acid and 80% (*v*/*v*) acetonitrile was used as both extraction solvent and diluent, and its combination with hexane in a partitioning step effectively removes proteins and fatty materials, meeting the requirements for detection by mass spectrometry.

### 3.2. Method Validation

Under the optimized mass spectrometry conditions and sample pretreatment methods, the LOD and LOQ for the five BAs are presented in Table 2. Among these, HIS exhibited the lowest LOD and LOQ, reaching 1.5 mg kg^−1^ and 4.0 mg kg^−1^, respectively, which are significantly below the allowable lowest limits of 50 mg kg^−1^ (FDA) for HIS in aquatic products. This satisfies the requirements for HIS eligibility fast screening and quantification analysis for quality evaluation using BAs as markers.

The linearity of the method was further investigated across concentrations ranging from 0.2 or 0.5 μg mL^−1^ (equivalent to LOQ in samples) to a maximum of 10 μg mL^−1^ for each analyte. The results demonstrated good linearity, with R^2^ values ranging from 0.9923 to 0.9961 (See also Table 2).

The accuracy and precision were further evaluated via a spike recovery experiment with three fortified levels and six replicates at each level. Because the background contents of SPD and SPM in the samples are relatively high and mostly above the LOQ, it is difficult to prepare spiked samples near the LOQ level; So, the lowest fortification level of SPD and SPM was set at 50 mg/kg in the experiment. The results are shown in Table 3. The recoveries ranged from 84.26% to 106.6%, with relative standard deviations (RSDs), the intra-day repeatability, between 4.56% and 13.84%, indicating that the method exhibits good accuracy and precision. Since BAs are stable, the instrument was recalibrated before each batch of sample analysis, and in addition, the stable isotope-labeled BA (HIS-d4) was adopted as the internal standard for overcoming matrix effects and operational errors. Accordingly, the evaluation of precision using only intra-day replicates is sufficient for method validation, and inter-day precision was therefore not conducted in this study.

**Figure 3:**
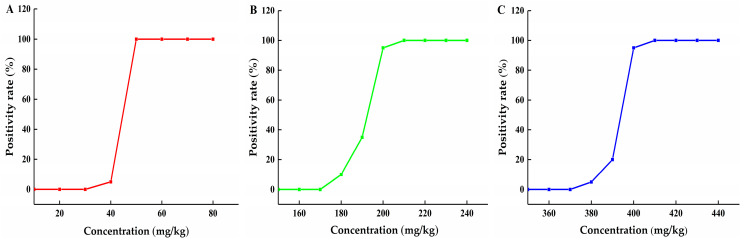
Positive rate detection curves of HIS in the fish matrix at three concentrations of concern. (**A**) Concentration of concern at 50 mg kg^−1^; (**B**) Concentration of concern at 200 mg kg^−1^; (**C**) Concentration of concern at 400 mg kg^−1^.

Regarding fish products, HIS is currently the only BA with established safety limits; however, these limits vary across countries. The U.S. FDA sets a limit of 50 mg kg^−1^, while China differentiates between low-histamine and high-histamine fish, with limits of 200 mg kg^−1^ and 400 mg kg^−1^, respectively. Therefore, the primary application of this method is rapid eligibility screening for histamine. For evaluating the performance of this eligibility screening method, three decision thresholds were calculated with the typical three safety limit concentrations of HIS mentioned above as the concentrations of concern, and the threshold values calculated were 49.35, 195.8, and 396.9 mg kg^−1^, respectively. With threshold values, the positive detection rate curves were constructed, and the minimum reliable detection concentration for positive samples (with a detection rate ≥95%) and the maximum reliable detection concentration for negative samples (with a positive detection rate ≤ 5%) were determined. The results, as shown in Figure 3, indicate that unreliable determinations are confined to a narrow concentration range from the maximum reliable detection concentration for negative samples to the minimum reliable detection concentration for positive samples, which were 40–50 mg kg^−1^, 180–200 mg kg^−1^ and 380–400 mg kg^−1^, respectively; the results demonstrated that this method is well-suited for the eligibility fast screening needs.

### 3.3. Practical Applications

With the method developed, BAs were detected in nine actual commercially available cold-chain fish products, and the detection results were re-verified using HPLC-MS/MS [29], as shown in Table 4. The results indicated that all nine samples tested negative (with a concern concentration of 50 mg/kg) for histamine, and the correctness of the determination results could be confirmed by HPLC-MS/MS. Only a few samples showed SPD and SPM levels exceeding the method’s LOQ, and these results were not significantly different from those obtained by the HPLC-MS/MS method (*p* ≥ 0.05). All the above indicate that the established method is feasible.

## 4. Conclusions

This study established a novel method for the rapid detection of five BAs, including HIS, TYR, CAD, SPD, and SPM, in cold-chain fish products with portable ion trap mass spectrometry with nano-electrospray ionization. The developed method features simple sample preparation, requires no derivatization or chromatographic separation, low cost and offers easy operation and rapid analysis with satisfactory accuracy and precision; It meets the requirements for BA safety detection and quality evaluation using BAs as markers in the cold-chain fish products. For HIS, which has safety limits in fish, the method could be successfully applied for the eligibility fast screening. The study provided a valuable methodological reference for the rapid detection of BAs in food.

## Figures and Tables

**Table 1:** Analytical parameters of five BAs and the internal compound HIS-d4.

Item	HIS	HIS-d4	CAD	TYR	SPD	SPM
Ionization mode	ESI+	ESI+	ESI+	ESI+	ESI+	ESI+
Ionization Voltage (V)	2500	2500	2000	2500	2000	2500
Injection time (s)	60	60	40	60	60	60
Scan range (*m*/*z*)	20–250	20–250	20–250	20–250	20–250	20–250
Collision gas	Air	Air	Air	Air	Air	Air
Precursor ion (*m*/*z*) and Collision energy (eV)	112; 0	116; 0	103; 0	138; 0	146; 0	203; 0
Product ions (*m*/*z*) and Collision energy (eV)	95; 2	99; 2	86; 1.5	121; 2	129; 2	129; 2.5

**Table 2:** Linearity, Limit of detection (LOD) and Limit of quantification (LOQ).

BA	LOD (mg/kg)	LOQ (mg/kg)	Linear Equation	Range (μg/mL)	R^2^
HIS	≤1.5	≤5.0	*y* = 0.4370*x* + 0.0224	0.2~10	0.9961
CAD	≤4.0	≤12.5	*y* = 0.0659*x* + 0.0053	0.5~10	0.9936
TYR	≤4.0	≤12.5	*y* = 0.5616*x* + 0.0425	0.5~10	0.9923
SPD	≤4.0	≤12.5	*y* = 0.2063*x* + 0.0332	0.5~10	0.9959
SPM	≤4.0	≤12.5	*y* = 0.3691*x* + 0.0769	0.5~10	0.9932

*y* is the peak area ratio of each BA to HIS-d4; *x* is the content ratio of each BA to HIS-d4.

**Table 3:** The results of the spiked recovery experiment (*n* = 6).

BA	Add Level (mg kg^−1^)	Recovery (%)	RSD (%)
CAD	12.5, 100, 200	85.36, 96.85, 92.41	12.53, 6.78, 5.68
HIS	5, 100, 200	85.44, 99.10, 94.28	9.62, 4.56, 4.74
TYR	12.5, 100, 200	97.47, 88.41, 103.6	11.26, 8.30, 6.69
SPD	50, 100, 200	84.26, 98.64, 97.49	12.86, 9.42, 8.87
SPM	50, 100, 200	104.8, 106.6, 88.75	13.84, 5.76, 8.02

**Table 4:** Determination results of 5BAs in actual cold-chain fish products with the portable ion trap mass spectrometry and HPLC-MS/MS.

Sample	Content (mg kg^−1^)				
	HIS	CAD	TYR	SPD	SPM
Salmon	ND ^a^	<LOQ	ND	<LOQ	18.87 ± 3.03
	0.96 ± 0.04 ^b^	6.12 ± 0.02	1.31 ± 0.10	10.80 ± 0.76	21.63 ± 1.77
Mackerel	ND	<LOQ	ND	12.79 ± 1.48	ND
	0.97 ± 0.08	5.96 ± 0.05	1.18 ± 0.09	10.75 ± 0.50	9.36 ± 0.82
Yellow Croaker	ND	<LOQ	ND	ND	17.57 ± 0.87
	1.01 ± 0.04	6.38 ± 0.01	1.20 ± 0.01	7.76 ± 0.54	19.43 ± 3.06
White pomfret	ND	44.64 ± 3.27	<LOQ	ND	ND
	1.41 ± 0.07	47.69 ± 0.26	7.90 ± 0.19	13.23 ± 0.24	16.96 ± 1.11
Chilled sea bass	ND	<LOQ	ND	14.04 ± 1.85	26.85 ± 2.73
	1.41 ± 0.57	6.35 ± 0.30	1.38 ± 0.03	16.49 ± 1.29	31.78 ± 1.63
Codfish	ND	<LOQ	ND	<LOQ	<LOQ
	0.96 ± 0.01	6.42 ± 0.25	0.99 ± 0.01	6.95 ± 0.56	8.78 ± 0.63
Balsa	ND	<LOQ	ND	<LOQ	ND
	0.99 ± 0.03	5.69 ± 0.10	1.06 ± 0.01	5.76 ± 0.57	3.31 ± 0.45
Pacific saury	ND	<LOQ	ND	16.03 ± 1.16	20.31 ± 3.09
	1.23 ± 0.18	6.97 ± 0.34	1.14 ± 0.04	18.10 ± 0.58	22.09 ± 0.96
Spanish mackerel	ND	<LOQ	ND	16.68 ± 2.82	17.88 ± 1.68
	1.26 ± 0.04	11.45 ± 0.16	2.04 ± 0.07	17.70 ± 0.27	16.78 ± 0.60

ND means not detected. ^a^ Portable ion trap mass spectrometry analysis results. ^b^ HPLC-MS/MS analysis results.

## Data Availability

The data that support the findings of this study are available from the corresponding author upon request.
